# Variations in Screening Adenoma Detection Rate by Specialty of Physicians in a Predominately African American Population

**DOI:** 10.7759/cureus.6003

**Published:** 2019-10-26

**Authors:** Shanker Kundumadam, Maliha Naseer, Zaid Kaloti, Wissam Kiwan, Pradeep R Kathi, Hala Nas, Paul H Naylor, Omar Al-Subee

**Affiliations:** 1 Internal Medicine, Wayne State University School of Medicine, Detroit, USA; 2 Gastroenterology, Wayne State University School of Medicine, Detroit, USA; 3 Internal Medicine / Gastroenterology, University of Arizona, Tucson, USA; 4 Gastroentoerlogy, Wayne State University School of Medicine, Detroit, USA

**Keywords:** colon cancer screening, adenoma detection rate, colonoscopy

## Abstract

Background: Screening colonoscopy aims to interrupt the adenoma-carcinoma sequence by removing all precancerous adenomatous polyps. Adenomatous polyp detection rate (ADR) can vary between endoscopists as well as between race, age, and risk of colorectal cancer (CRC). The purpose of this study was to compare ADR among academic gastroenterologists (A-GI), non-A-GI, and surgeons for endoscopies performed in the same endoscopic suite of a large medical center with a predominately African American (AA) population.

Methods: All screening colonoscopies performed in 2014 for patients aged 62-76 years were identified using the electronic medical records data. Patients with average risk and high risk of CRC defined as having a 'personal history of polyps' or 'family history of CRC', and history of ulcerative colitis and Fecal Occult Blood Test/Fecal Immunochemical Test (FOBT/FIT) positivity were included. Patients with incomplete colonoscopy (defined as failing to achieve cecal intubation or poor preparation) and unrecovered tissue biopsy were excluded. ADR was calculated for three groups of endoscopists: A-GIs, non-A-GIs, and surgeons.

Results: A total of 573 screening colonoscopies was analyzed. The endoscopists comprised five A-GIs, eight non-A-GIs, and six surgeons. The majority of patients were of AA decent (71%), female (54%) with an average age of 66 years. Patients classified as average risk comprised 79% of the population. Most of the colonoscopies were performed by A-GI (n=339), followed by non-A-GI (n=144), and surgeons (n=90). The ADR for A-GI was 50% as compared to 32% for non-A-GI (p<0.001) and 25% for surgeons (p<0.001). Also, A-GI were more likely to identify ≥3 adenomas during screening colonoscopies. Significant differences were observed (p<0.001) in the mean time of colonoscopy for A-GI (30 mins) non-A-G (14 mins), and surgeons (18 mins).

Conclusion: Significant variation in the ADR between endoscopists belonging to different specialties were observed. Although all appear to achieve acceptable ADR (ie at least 25 for men and 15 for women), academic gastroenterologists had better performance than non-academic GI and surgeons. This may be explained by a significantly longer average duration of procedures for the highest ADR group.

## Introduction

Colorectal cancer (CRC) screening is effective in reducing the occurrence and mortality of colorectal disease by identification and removal of adenoma and precursor adenoma lesions by the endoscopist [1-3]. The effectiveness of this approach has been demonstrated in multiple large population studies where the detection of adenomas as defined by the number of patients who have an adenoma (adenoma detection rate (ADR)) is correlated with reduction of interval cancers [4-10]. Thus ADR has been identified as the most important quality measure to assess colonoscopies. While there are also additional indices used as quality measures such as withdrawal time, cecal intubation rate, and polypectomy removal, ADR remains the most useful benchmark parameter for the effectiveness of endoscopists in decreasing the risk of patients for interval cancers [6-10].

Interval colorectal cancer is defined as colorectal cancer diagnosed within five years of a negative screening colonoscopy. Although ADR is an independent predictor of the risk of interval colon cancer, there can be variation in ADRs based on age, gender and race of patients, quality of bowel prep, etc., which can act as confounding factors in defining the ADR performance of endoscopists [11]. On the other hand, studies have shown that risk of interval cancer is greater in patients who had procedures done by endoscopists with ADR less than 20% compared to ones done by endoscopists with ADR more than 20% [8]. In patients who have had a negative colonoscopy, those who had the procedure done by gastroenterologists were less likely to develop interval cancer in comparison to non-gastroenterologists [12].Studies have looked up at polypectomy rates of gastroenterologists vs. surgeons and found it to be lower for surgeons [13]. Additional studies have compared physician characteristics and potential interventions to define variables that could be modified to enhance ADR [14-21].^ ^What has not been systematically studied is a comparison of ADR between academic gastroenterologists, non-academic gastroenterologists, and surgeons who are seeing similar patient populations with similar gender distribution and age.

The objective of our study was to look at screening colonoscopies in a large medical center and compare the ADRs between academic gastroenterologists with an appointment in the medical school and fellow training responsibility, non-academic gastroenterologists in private practice, and surgeons. As all the above mentioned physicians did the procedures in the same endoscopic facility, it would avoid some of the confounding factors like the quality of the equipment, expertise of the supporting staff, etc.

This article was presented as a poster. (Poster: Nasser M, Kaloti Z, Kiwan W, Nas H, Naylor P, Al-Subee O. P2041 - Variations in Adenomatous Polyps Detection During Screening Colonoscopy by Specialty and Practice Setting of Endoscopists Performed in a Predominately African American Population. Program No. P2041. World Congress of Gastroenterology at ACG2017 Meeting Abstracts. Orlando, FL: American College of Gastroenterology. Oct 17, 2017. https://eventscribe.com/2017/wcogacg2017/ajaxcalls/PosterInfo.asp?PosterID=116204&efp=S1lVTUxLQVozODMy&rnd=0.7653571)

## Materials and methods

This was a retrospective study conducted in a large medical center. All screening colonoscopies done in the year 2014 among patients in the age group 62 to 75 years were selected using the electronic medical records of the medical center. All the procedures were done at the same endoscopy suite thus eliminating the variations in observations due to the difference in the equipment. Colonoscopies were performed by three groups of physicians: academic gastroenterologists (A-GI), non-academic gastroenterologists in private practice (non-A-GI), and surgeons.

Statistical analysis was performed using JMP software (JMP Version:14.0.0, by SAS Institute Inc, NC, USA). Significance was defined using Student’s t-test for continuous variables and Pearson chi-square analysis for character variables. The patients were categorized based on their age, gender, race and were also classified into either average risk or high risk. High risk category included patients with a personal history of colonic polyps, family history of colon cancer and those patients with a history of ulcerative colitis. All other patients other than the above mentioned high risk category was classified as average risk patients.

Those procedures without adequate bowel prep as noted in the record and those without cecal intubation were excluded from the study. Procedures with unrecovered tissue biopsy were also excluded. ADR was calculated as the number of patients with at least one histologically confirmed adenoma divided by the total number of colonoscopies performed.

## Results

A total of 573 screening colonoscopies were analyzed. Most patients were of African American (AA) decent (n=406, 71%). The average age of the patient population was 66; 54% (n=309) of these patients were females. Average risk patients comprised 79% (n=452) of the patient population. The majority of the colonoscopies done in the institution during this time was done by the A-GI (n=339, 59% ) compared to 144 in the non-A-GI group and 90 in the surgeons (Table 1). The number of endoscopists was similar in each group (A-GI=5, non-A-GI=8, and S=6). The majority of the patients in the A-GI and the non-A-GI were African Americans (77%, n=261, and 71%, n=102, respectively), whereas the surgeon group had a near similar distribution of patients by race (52% African American). Both academic and non-academic GI had more female patients than males (57% and 59%, respectively), whereas surgeons had predominantly male patients (70%). Univariate analysis confirmed A-GI and non-A-G had similar patients while the patient distribution in the surgeon group was different.

**Table 1:** Demographic and performance factors that could cause variation in the ADR of screening colonoscopy by specialty of physicians. Academic GI - Academic Gastroenterologist, Non-Academic GI - Non-Academic Gastroenterologist, NS - Not Statistically Significant, ADR - Adenomatous Polyp Detection Rate

Variables	Academic GI	Non-Academic GI	Surgeons	Overall p-value	Academic vs Non-Academic p- value	Academic vs Surgeon p- value
Race (%)						
African American	76.99	70.83	52.22	<0.001	NS	<0.001
Non-African American	23.01	29.17	47.78			
Gender (%)						
Male	42.77	40.28	70	<0.001	NS	<0.001
Female	57.23	59.72	30			
Risk (%)						
Average	82	75.69	72.22	<0.001	NS	<0.05
High	18	24.31	27.78			
Age (years)	66	68	67	<0.001	<0.001	<0.005

**Figure 1:**
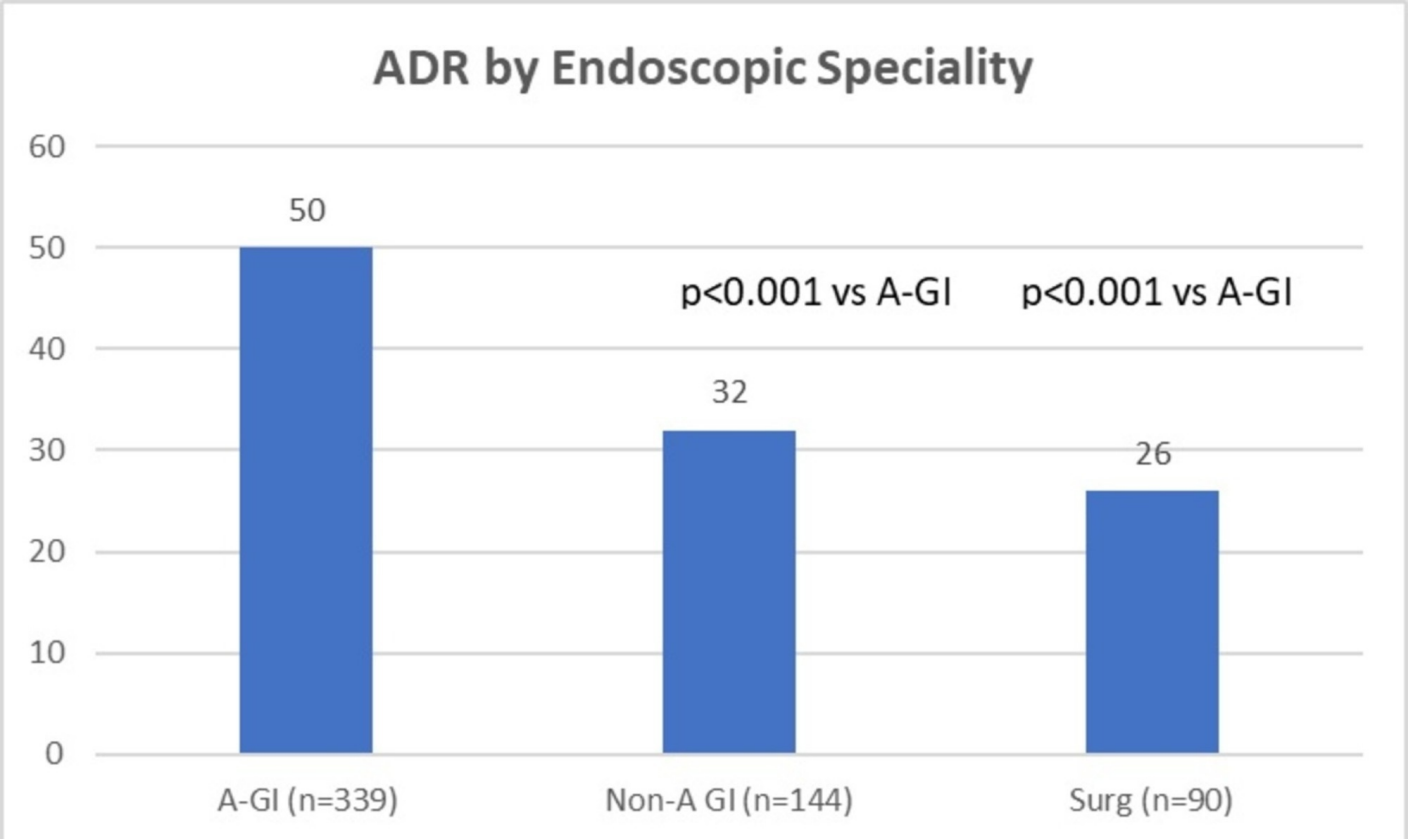
Adenomatous polyp detection was significantly better for Academic GI (A-GI) than for Non-Academic GI (GI) and Surgeons (Surg). There was no difference between Non-A-GI and Surgeons (p=0.29). The number of patients in each group is in parenthesis.

The adenoma detection rate was significantly better for A-GI compared to non-AGI and surgeons (49.8 vs. 31.9 vs 25.5; p-value - <0.0001) (Figure 1). There was no difference between the non-A-GI and surgeons (p=0.29). A-GI were also more likely to identify ≥3 adenomas during screening colonoscopies (academic= 16% vs non-academic=4% vs surgeons=7%; p<0.001).

With respect to ADR in subsets of patients by each of the specialties, regardless of race (AA vs non-AA), gender, and risk, the A-GI physicians consistently had the greater ADR as compared non-A-GI and surgeons (Table 2). With respect to ADR within groups of patients with the same class of endoscopists, it was not statistically significantly different with respect to the rates of adenomas detected (ADR) with the exception of race for patients of surgeons (36 AA vs 14 for non-AA) and gender for patients of academic GI (58 for male vs 44 for females). The most striking difference between the three groups was in the time to perform the colonoscopy, which was significantly longer for A-GI (31 min) as compared to non-A-GI (14 min) and surgeons (18 min). As expected, patients with adenomas required a longer time to complete their procedure. For patients who had no adenomas, the colonoscopy time reflected that for the total patient population of the specialties (A-GI=26.9 min vs non-A-GI=12.8 min vs surgeons=15.6 min). Since the presence of a fellow could impact the colonoscopy time, we also evaluated the time in patients without an adenoma and it was no different whether a fellow was present (27 min) or not (26 min).

**Table 2:** ADR as function of variables for each of the classes of endoscopists. ADR - Adenomatous Polyp Detection Rate

Variables	Academic GI	Non-Academic GI	Surgeons
African American	ADR=52	ADR=30	ADR=36*
Non-African American	ADR=44	ADR=35	ADR=14
Male	ADR=58*	ADR=38	ADR=22
Female	ADR=44	ADR=28	ADR=33
Average risk	ADR=48	ADR=28	ADR=31
High risk	ADR=61	ADR=43	ADR=12
Time for colonoscopy	30.6 min**	13.9 min**	18.3 min**
Time with adenoma	34.4 min***	16.4 min***	25.8 min***
Time with no adenoma	26.9 min	12.7 min	min
*P<0.05 for patient with adenoma vs no adenoma **p<0.001 for academic vs non-academic and academic vs surgeons; NS for non-academic vs surgeons *** p<0.005 for patients with adenoma vs non adenoma			

## Discussion

There have been several studies in the literature which compared the variation of ADR based on the specialty of the physicians doing the procedure and other factors such as training and experience [12-21]. To our knowledge, our study is the only one that looked at the variation in ADR between academic gastroenterologists, non-academic gastroenterologists, and surgeons. Academic gastroenterologists were defined as those in an academic fellowship program where many of the procedures are done with gastroenterology fellows. In our institution, all three specialties do the procedure in the same endoscopy suite. This eliminates some of the confounding technical factors and the contribution by the non-physician support staff. There were, however, differences in patient-related factors between the three groups. Both academic and non-academic GI had more African American patients, and an increase in their ADR based on the higher prevalence of adenomas in African American patients was possible. This possibility is countered by the inclusion of only older patients (<62 years) where the variation due to race declines and the comparison of ADR by race in the three groups with the observation that the differences in ADR were present for both races. It is important to note that surgeons had more male patients and high-risk patients, both of which should contribute to a higher adenoma detection rate whereas the relative rates remained the same regardless of gender or risk.

As the only consistent difference between procedures performed by the three groups was the time of colonoscopy, it appears likely that the academic GI outperformed both nonacademic GI and surgeons in terms of ADR due a longer and possibly more thorough examination of the colon [17-18]. This can be equated to a longer withdrawal time, which has been equated with a higher ADR in a number of studies [17-21]. Asfaha et al. noted that gastroenterology trainees had more colonoscopy volume during their training compared to surgery trainees and this could certainly hold true for the surgeons but we postulate it is unlikely for the non-academic GI [19]. 

A large number of patients seen in one endoscopic suite is a major strength of the study. Also unique to this study is the comparison with non-academic GI in the same suite. The fact that the populations were slightly different between GI and surgeons could be construed as a weakness of the study but alternatively provides evidence that demographics may not play a significant role with respect to comparing ADR. The major weakness of our study is that the endoscopic reports for the majority of patients did not report the withdrawal time, so only total time for colonoscopy was available. We did correct for this by comparing colonoscopy time for procedures when no adenoma was present and by demonstrating that the presence or absence of a fellow in training had no impact on the time.

## Conclusions

Overall the striking differences noted in the ADR between specialties certainly have some major implications. It is important that the patient be aware of the higher probability of missed neoplasms when the procedure is done by a specialty with low ADR. This is also relevant in terms of insurance reimbursements and cost-effective care. With respect to surgeon ADR, studies including ours have suggested the need for modifications in the structure of training for surgeons in order to better equip them to have a higher ADR. There have also been reports that recording the ADR of individual endoscopists and providing them with a comparison of their results with that of the average for an endoscopy suite is a useful intervention to improve ADR rates. Therefore managers of the endoscopic suites in multi-specialty settings should encourage a program to institute such a procedure.
